# Stock Market Returns and Clinical Trial Results of Investigational Compounds: An Event Study Analysis of Large Biopharmaceutical Companies

**DOI:** 10.1371/journal.pone.0071966

**Published:** 2013-08-07

**Authors:** Thomas J. Hwang

**Affiliations:** Faculty of Arts and Sciences, Harvard University, Cambridge, Massachusetts, United States of America; Tel Aviv University, Israel

## Abstract

**Background:**

For biopharmaceutical companies, investments in research and development are risky, and the results from clinical trials are key inflection points in the process. Few studies have explored how and to what extent the public equity market values clinical trial results.

**Methods:**

Our study dataset matched announcements of clinical trial results for investigational compounds from January 2011 to May 2013 with daily stock market returns of large United States-listed pharmaceutical and biotechnology companies. Event study methodology was used to examine the relationship between clinical research events and changes in stock returns.

**Results:**

We identified public announcements for clinical trials of 24 investigational compounds, including 16 (67%) positive and 8 (33%) negative events. The majority of announcements were for Phase 3 clinical trials (N = 13, 54%), and for oncologic (N = 7, 29%) and neurologic (N = 6, 24%) indications. The median cumulative abnormal returns on the day of the announcement were 0.8% (95% confidence interval [CI]: –2.3, 13.4%; P = 0.02) for positive events and –2.0% (95% CI: –9.1, 0.7%; P = 0.04) for negative events, with statistically significant differences from zero. In the day immediately following the announcement, firms with positive events were associated with stock price corrections, with median cumulative abnormal returns falling to 0.4% (95% CI: –3.8, 12.3%; P = 0.33). For firms with negative announcements, the median cumulative abnormal returns were –1.7% (95% CI: –9.5, 1.0%; P = 0.03), and remained significantly negative over the two day event window. The magnitude of abnormal returns did not differ statistically by indication, by trial phase, or between biotechnology and pharmaceutical firms.

**Conclusions:**

The release of clinical trial results is an economically significant event and has meaningful effects on market value for large biopharmaceutical companies. Stock return underperformance due to negative events is greater in magnitude and persists longer than abnormal returns due to positive events, suggesting asymmetric market reactions.

## Introduction

Research and development (R&D) is a hallmark of innovative biopharmaceutical companies. The stakes for these R&D activities are high due not only to their contribution to firm profitability and competitiveness but also to their role in potentially relieving suffering and curing sickness.

A number of industry commentators have argued that the biopharmaceutical industry faces a R&D ‘productivity crisis.’ [1], [2] There is some evidence to suggest that development risks, which refer to the likelihood that the R&D process will be terminated due to safety, efficacy, or commercial concerns, remain high. [3], [4], [5] Late-stage clinical studies (Phase 2–3) are also the most expensive stage of the drug development process, making attrition during this stage particularly problematic for management and investors. [6], [7] The costs of Phase 3 trials alone are estimated to account for up to 40% of total R&D costs. [8].

Given the risks, uncertainty, and difficulty associated with developing new medicines, the release of clinical trial results is an important and closely anticipated inflection point in the R&D lifecycle. As the stock price of a firm represents an assessment of its current and future earnings capacity, these announcements are expected to have positive or negative effects on the firm’s market valuation. There has been increasing attention paid by the medical and scientific communities to this and other measures of the financial performance of biopharmaceutical companies. For example, some have suggested that prescribers could monitor stock prices to ascertain early warnings about drug safety. [9] Some physicians and scientists are also involved in a range of financial activities, ranging from consulting and investment advice to employment by brokerages and hedge funds to personal trading. [10] Expert knowledge of clinical trials can be lucrative, [11], [12] and the potential for insider trading is an ever-present risk. [13].

Previous research on R&D and market valuation has largely focused on capital expenditures or product development outcomes, such as drug approvals and patents. Some have observed positive share price responses to increased R&D spending despite lower earnings. [14] Others have found that financial market losses from product development failures are larger in magnitude than gains from successes. [15], [16] There has been limited empirical work examining how and to what extent the stock market values measures of R&D, such as clinical trials, for investigational compounds. A pair of studies, one on oncology drugs [17] and another on biotechnology products [18], found that there are measurable changes in company stock prices before important trial announcements and significant differences in stock price changes between companies with a successful product versus those with an unsuccessful product.

The primary aim of this study was to investigate the impact of unforeseen clinical research results on stock market returns for biopharmaceutical companies that obtain capital through the public equity market, i.e. publicly traded companies. To measure the market value effects of our sample of clinical R&D events, we used event study methodology, which is widely used in financial economics to measure abnormal returns from a given stock after adjusting for normal market performance [19], [20] though its application to the clinical-scientific literature is relatively new. [21], [22] Specifically, we proposed using a short horizon event study, narrowing the time period of analysis to two days before and after an event, to isolate the market reaction to that event. Therefore, barring confounding events, this method captures the natural experiment that occurs in stock returns due to new information, and more accurately accounts for financial performance than changes in stock prices alone. We defined the following hypotheses relating to the public market’s valuation of and reaction to clinical development events:

H1 (Null): There is no difference between abnormal stock market returns immediately before or after announcements of clinical trial results and zero.

H1.1 (Alternative): Positive development events are associated with significant and positive share price changes.

H1.2 (Alternative): Negative development events are associated with significant and negative share price changes.

H2 (Null): There are no differences in abnormal stock market returns due to positive versus negative announcements.

H2 (Alternative): The abnormal stock market returns due to negative announcements are significantly larger than those for positive events.

## Methods

### Sample Selection

Our event study focused on the R&D activities of large publicly traded biopharmaceutical companies in the U.S. from January 1, 2011 to May 1, 2013. A ‘large biopharmaceutical company’ was defined as a biotechnology or pharmaceutical company with revenues from branded pharmaceutical products, such as drugs and biologics, representing at least 50% of total gross revenues and in excess of US$5 billion, as of 2012. Descriptive statistics of the seven firms meeting these criteria are provided in Table 1.

**Table 1:** Descriptive statistics of large biopharmaceutical firms.

Firm	Ticker	2012 Revenues		
		Total	Pharma	%
Amgen	AMGN	17,265	17,265	100
Biogen Idec	BIIB	5,517	5,517	100
Bristol-Myers Squibb	BMY	17,621	17,621	100
Eli Lilly & Co.	LLY	22,603	20,567	91
Gilead Sciences	GILD	9,703	9,703	100
Merck	MRK	47,267	40,601	86
Pfizer	PFE	58,986	40,979	69

**Notes**: All data represent 2012 calendar year financials. Pharmaceutical segment revenues (“Pharma”) represent the amount and proportion of total revenues from branded (i.e. non-generic) pharmaceuticals. **Sources**: Company filings with the Securities and Exchange Commission and Capital IQ.

### Data Collection and Extraction

For our sample of firms, we searched Factiva for news articles and public announcements of results from Phase 1, 2, and 3 trials of investigational compounds. The key words for the search included: ‘clinical trials,’ ‘studies,’ ‘research,’ ‘Phase 1,’ ‘Phase 2,’ ‘Phase 3,’ ‘science,’ and ‘scientific evidence.’ We included the first announcement that signaled a positive or negative result, and excluded announcements for studies of compounds that had been approved by the U.S. Food and Drug Administration (FDA) or European Medicines Agency (EMA) as of May 1, 2013. We also manually collected and reviewed press releases, transcripts of quarterly earnings calls, and other public statements from company websites. Additional identified announcements were merged with our search results, following exclusion of non-relevant or duplicate citations.

Data on study characteristics, outcome measures, and results were extracted from the announcements in our merged search results using a pre-defined extraction form. This form captured: company, publication date, phase of trial, study design, observation period, number of patients treated, number and type of treatment arms, investigational compound used, indication(s), primary and secondary endpoints, whether results were interim (or ‘top-line’) or final, whether the primary and/or secondary endpoint(s) were met, and whether the data were (or due to be) presented at a conference or published in a peer-reviewed journal. Based on the extracted data, the announcements were ordered chronologically and assigned *a priori* expected signs. An announcement was coded as positive if the firm reported meeting the primary endpoint or, in the case of interim results, efficacy or safety data meriting the firm’s regulatory submission or initiating the next stage of clinical development. Negative events involved results failing to meet pre-specified outcome measures and discontinuations of studies due to safety, efficacy, or other reasons. The coding of expected signs was validated by review of the original text.

### Event Study Methodology

The standard short horizon event study methodology for market-adjusted residual returns relies on the assumption of efficient capital markets—that is, stock prices reflect the continuum of new information received and processed by market participants. As all of the firms in our sample are large and listed primarily on U.S. stock exchanges, we did not have to account for thin market trading volumes or other possible violations of event study assumptions.

First, we defined the event window as two and one trading days before and after the event date, defined as day 0, and reference window as 300 trading days (–310 to –10) before the date. Reference windows of similar length have been used widely in the literature. [21]–[22] To reduce the likelihood of confounding events and bias, we excluded events in our sample with overlapping ± 2 day windows with material firm-level events. Potentially confounding events included: other announcements in our sample; regulatory, legal, financial (e.g. mergers and acquisitions, licensing agreements, etc.) and corporate management (e.g. resignations) decisions; and announcements for studies of approved products.

For each firm *i* at time *t* during the estimation window, the coefficients α and β of the market model were estimated by OLS using stock price data from Bloomberg Professional. Formally, the estimation equation is:
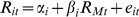
(1)


where R*_it_* is the stock return and R*_Mt_* is the normal market return. The value of the S&P 500 stock index was used as the market portfolio.

Next, we calculated the daily abnormal return (AR*_it_*) by comparing the stock return at time *t* during the event window to the expected market return if the event had not occurred:
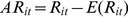
(2)


Finally, cumulative abnormal returns (CAR) during the event window (t_1_, t_2_) are given by:
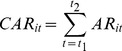
(3)


Firm-level and aggregate CAR were calculated for the time-symmetric (–2, +2) and (–1, +1) windows. To examine the immediate effects of the announcement on stock returns and to account for potential information leakage and/or latecomers to the event, we also calculated CAR for the periods (–2, 0), (–1, 0), and (–2, +1). For these five sub-windows, we also calculated the proportions of events with the same signs (positive or negative) as expected.

### Statistical and Sensitivity Analyses

Given the small sample size, we used the nonparametric one-sample Wilcoxon signed-rank test to test the null hypothesis that the median CAR for a given window is equal to zero. The Wilcoxon signed-rank test considers both the sign and magnitude of abnormal returns and does not assume normality or infer the value of any population parameter. This approach, though less common in the literature than parametric *t* statistic tests, has been documented to be preferable to other test methods with respect to event clustering, increases in variance on the event day, and Type I errors (or the incorrect rejection of null hypotheses). [23], [24], [25], [26] To compare the proportion of CAR signs at different times during the event window, we also used the binomial test.

As a measure of the robustness of our results, we conducted sensitivity analyses using a shorter estimation reference window of 30 trading days before and after the announcement date. The Mann-Whitney-Wilcoxon test was used to compare abnormal returns by firm type (biotech vs. pharmaceutical), proportion of R&D spending relative to total revenues, market capitalization, indication, and trial phase. We also used regression models with and without bootstrap resampling to test for potential interactive effects. Statistical analyses were performed using Stata (version 12.0, Stata Corp, College Station, TX, USA), with a two-sided α  = .05.

## Results

**Figure 1:**
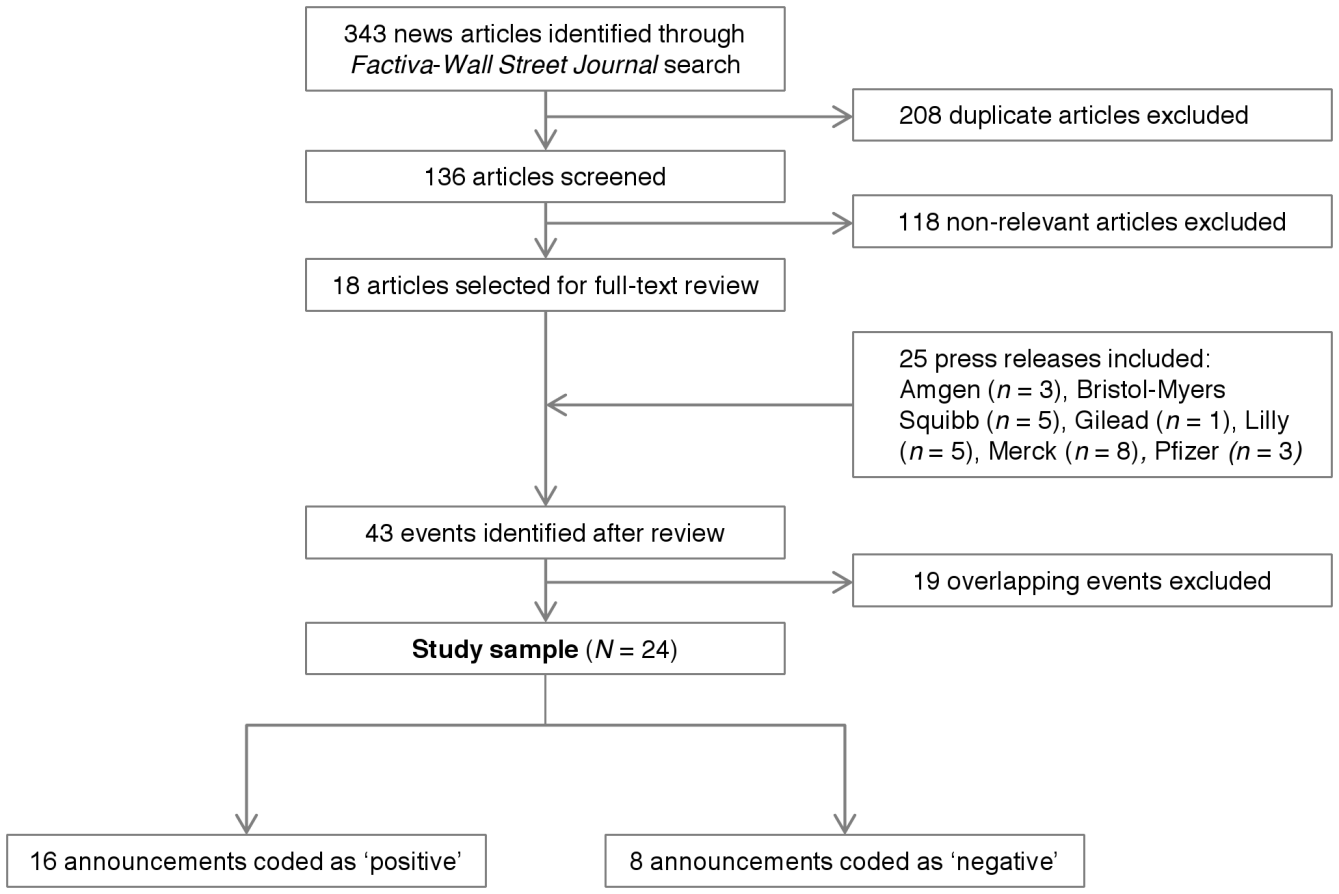
Sample selection flowchart.

Our sample comprised 24 public results announcements relating to 23 clinical trials registered on ClinicalTrials.gov (Figure 1) and 24 unique investigational compounds. Two articles reported data on different combination regimens from a multipart Phase 2 trial of sofosbuvir (GS-7977) and ribavirin in hepatitis C genotype 1 patients. Table 2 shows the characteristics of our sample events. Of the 24 events, 16 (67%) were positive and eight (33%) were negative. Five (31%) of the positive announcements were for Phase 3 clinical trials, nine (56%) for Phase 2, and two (13%) for Phase 1. All of the negative announcements were for Phase 3 trials. There were an equal number of announcements from large pharmaceutical companies compared with biotechnology companies. Most of the selected events were for oncologic (N = 7, 29%) and neurologic (N = 6, 24%) indications. There were an additional three trials for hepatitis C, two for diabetes and hypertension, two for psoriasis, one on hemophilia B, one on HIV, one on rheumatoid arthritis, and one on ragweed allergies. Nine (38%) compounds were biologics.

**Table 2:** Summary table of selected clinical development events in 2011–2012.

Event	Date	Company	Expected Sign	Phase	Indication	Compound	Trial ID
1	3/19/2013	AMGN	Positive	3	Melanoma	Talimogene laherparepvec (OncoVEXGM-CSF)	NCT00769704
2	3/5/2013	GILD	Positive	2	HIV	GS-7340 (Tenofovir Alafenamide)	NCT01497899
3	2/7/2013	LLY	Negative	3	Rheumatoid arthritis	Tabalumab	NCT01202773
4	1/24/2013	BIIB	Positive	3	Multiple sclerosis	BIIB-017 (PEGylated Interferon Beta-1a)	NCT00906399
5	1/3/2013	BIIB	Negative	3	Amyotrophic lateral sclerosis	Dexpramipexole	NCT01281189
6	12/5/2012	PFE	Positive	2	Breast cancer	PD-0332991	NCT00721409
7	11/10/2012	GILD	Positive	2	Hepatitis C	GS-7977 (sofosbuvir) and GS-5885	NCT01260350
8	10/11/2012	PFE	Positive	3	Chronic noncancer pain	ALO-02 (oxycodone hydrochloride and naltrexone hydrochloride extended-release capsules)	NCT01428583
9	9/26/2012	BIIB	Positive	3	Hemophilia B	Recombinant Factor IX Fc Fusion Protein (rFIXFc)	NCT01027364
10	8/8/2012	AMGN	Negative	3	Metastatic adenocarcinoma of the pancreas	Ganitumab (AMG 479)	NCT01231347
11	8/6/2012	PFE	Negative	3	Alzheimer's disease	Bapineuzumab	NCT00574132
12	8/2/2012	BMS	Negative	3	Hepatitis C	BMS-986094	NCT01629732
13	7/11/2012	LLY	Negative	3	Acute schizophrenia	LY-2140023 (pomaglumetad methionil)	NCT01487083
14	7/2/2012	BMS	Positive	1	Metastatic castration-resistant prostrate cancer, Renal cell carcinoma, Metastatic melanoma, Non-small cell lung cancer	BMS-936558	NCT00730639
15	5/22/2012	LLY	Positive	2	Type 2 diabetes	LY-2189265 (dulaglutide)	NCT01149421
16	5/16/2012	AMGN	Positive	2	Acute lymphoblastic leukemia	Blinatumomab (AMG-103)	NCT01209286
17	3/28/2012	AMGN	Positive	2	Psoriasis	Brodalumab (AMG-827)	NCT00975637
18	3/28/2012	LLY	Positive	2	Psoriasis	Ixekizumab (LY-2439821)	NCT01107457
19	3/25/2012	AMGN	Positive	1	Hypertension	AMG-145	NCT01133522
20	3/5/2012	MRK	Positive	3	Allergies (Ragweed)	MK-3641 (Allergy Immunotherapy Tablet)	NCT01469182
21	2/3/2012	GILD	Positive	2	Hepatitis C	GS-7977 (sofosbuvir)	NCT01260350
22	12/22/2011	BMS	Negative	3	Hepatocellular carcinoma	BMS-582664 (brivanib)	NCT00825955
23	8/9/2011	BIIB	Positive	2	Multiple sclerosis	Daclizumab	NCT00390221
24	2/2/2011	BMS	Negative	3	Non-small cell lung cancer	Necitumumab	NCT00982111

**Note**: Sample announcements were manually matched with unique clinical trial identifiers provided by ClinicalTrials.gov, a public database.

### Stock Returns Analysis

**Figure 2:**
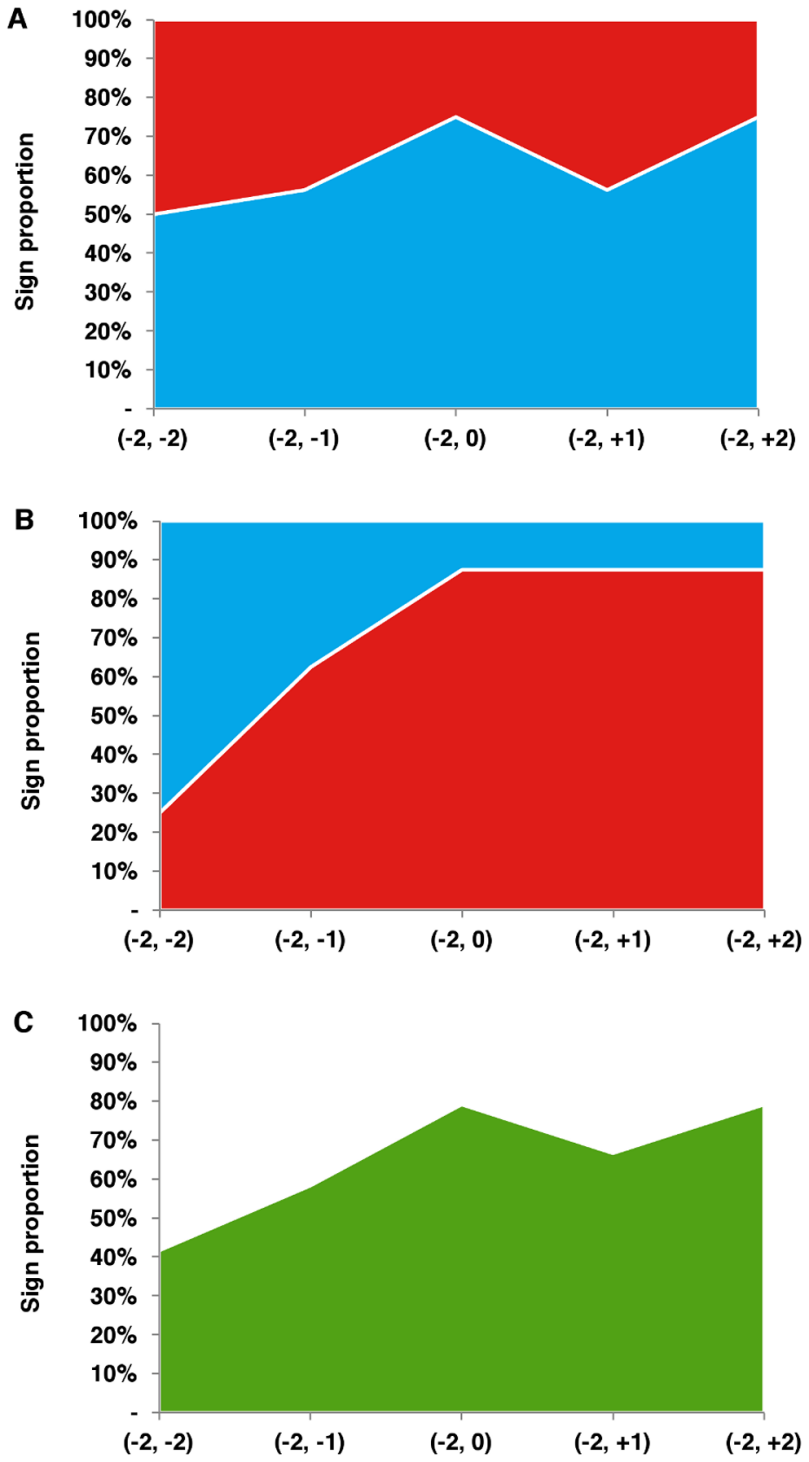
Sign of CAR (–2, *t*) stratified by positive and negative events. Notes: Panel A shows the proportion of CAR due to positive events with positive (blue) and negative (red) signs during the ± 2 trading day window. Panel B shows the proportions for negative events. Panel C displays the proportion of events with the same actual and expected CAR sign for the entire sample.

All positive events were associated with stock price increases during the ± 1 and ± 2 trading day event windows, whereas negative events were associated with stock price decreases. In the majority of cases, the sign of the cumulative abnormal returns (CAR) ± 2 days matched the expected *a priori* sign (Figure 2). For positive announcements, the CARs for 12/16 events were positive by market close of the day of the announcement, compared with 8/16 two trading days prior. There were a modest number of CAR sign reversals on the day after, with 9/16 events with positive CARs, though the difference in proportions was not statistically significant (P = 0.09). For negative announcements, the proportion of events with negative CARs increased steadily from –2 days to the day of the announcement. The proportion of events with negative CARs was significantly greater on day 0, +1, and +2 compared with –2 (P = 0.004).

**Figure 3:**
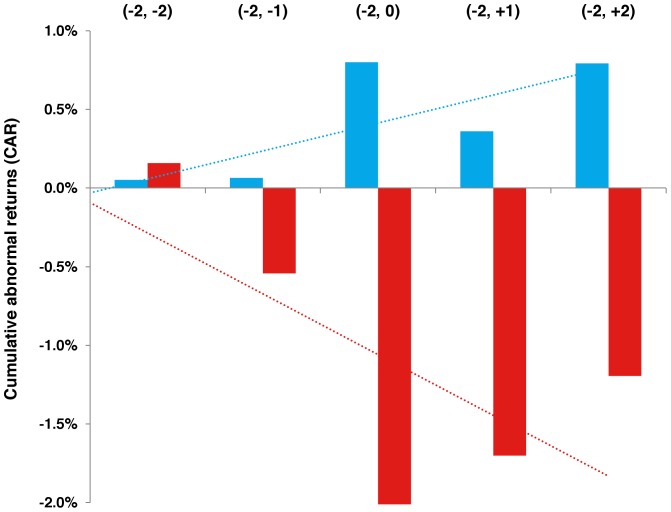
Median CAR (–2, *t*) for positive and negative events. Note: Median CAR during the ± 2 trading day window are shown for positive (blue) and negative (red) events.

Figure 3 depicts the median CARs for positive and negative announcements along the ± 2 day event window. The absolute value of CAR was greatest on the day of the announcement (–2, 0) for both positive and negative events. The median CAR (–2, 0) for positive events was 0.8% (95% confidence interval [CI]: –2.3, 13.4%), and the increase in CAR was statistically significant (P = 0.02). The median CAR for (–2, +1) and (–2, +2) remained positive, at 0.4% (95% CI: –3.8, 12.3%) and 0.8% (95% CI: –3.9, 12.2%) respectively, though the differences were not statistically significant (P = 0.33). For negative events, the median CAR (–2, 0) was –2.0% (95% CI: –9.1, 0.7%), which was statistically significant (P = 0.04). The median CAR for (–2, +1) and (–2, +2) were –1.7% (95% CI: –9.5, 1.0%; P = 0.03) and –1.2% (95% CI: –10.0, 1.0%; P = 0.04), respectively.

**Figure 4:**
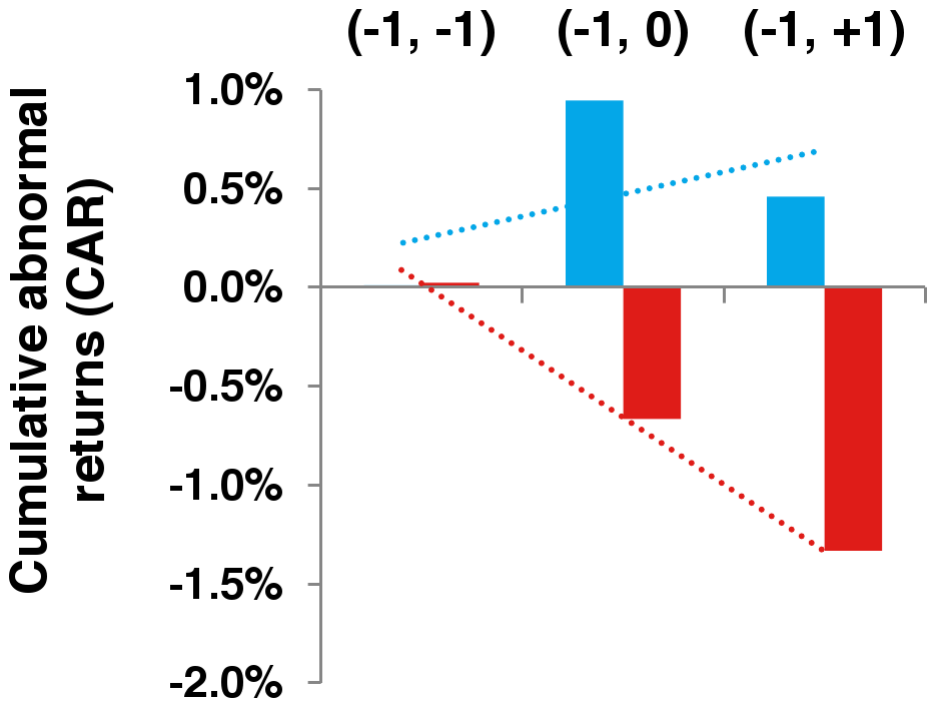
Median CAR (–1, *t*) for positive and negative events. Note: Median CAR during ± 1 trading day window shown for positive (blue) and negative (red) events.

To account for the possibility of information leakage, we also analyzed abnormal returns from a shorter window of ± 1 day relative to the date of announcement (Figure 4). Over the window (–1, 0), the median CARs were 0.9% (95% CI: –2.8, 13.5%; P = 0.02) and –0.7% (95% CI: –8.2, –0.02%; P = 0.01) for positive and negative events, respectively. Over the entire ± 1 day window, the CAR for positive events failed to reach statistical significance (median: 0.5%; P = 0.28). The median CAR (–1, +1) for negative events was –1.3% (95% CI: –9.1, 0.6%; P = 0.04).

### Sensitivity Analysis

Our results were robust to the use of an estimation period of thirty trading days before and after the event rather than three hundred preceding trading days (Figure S1). With the shorter reference window, the median CAR (–2, 0) for positive events was 0.7% (95% CI: –1.5, 13.5%; P = 0.02) and –1.4% (95% CI: –6.6, 1.0%; P = 0.09) for negative events. Over the entire ± 2 day window, significant CAR persisted for negative events (median: –1.4%; P = 0.04) but not for positive events (median: 0.6%; P = 0.20).

For both positive and negative events, there were no significant differences in CAR over the ± 2 day window between late-stage (Phase 3) and early-stage (Phase 2 and 1) announcements, between biotechnology and pharmaceutical firms, or by indication or trial phase. In a multiple regression stratified by event type with and without bootstrap resampling, these variables were not significant predictors of CAR.

## Discussion

In this paper, we evaluated the impact of R&D announcements on the financial performance of large U.S. biopharmaceutical companies, using event study methodology to link a novel dataset of results from clinical trials of investigational compounds with daily stock market returns. We found that both positive and negative announcements resulted in statistically significant changes in cumulative abnormal returns on the day of the announcement. The median decline in cumulative abnormal returns due to negative events was larger in magnitude than gains due to positive events, therefore rejecting our null hypotheses. We also found that the CAR due to negative announcements remained significantly negative through the entire two day window, whereas positive events were associated with stock price corrections the day after the event. Abnormal returns due to positive or negative announcements did not differ by indication, phase, or firm type (biotech versus pharmaceutical). In a *post hoc* sensitivity analysis, our results were robust to an alternative reference window.

Our results indicate that clinical development events have material economic implications, even for large biopharmaceutical companies with multidrug portfolios, and provide insight into the short-term behavior of the market in response to trial results. These findings confirm and extend previous scholarship on the significant market reactions to clinical trial results for biotechnology companies with few compounds in development. Moreover, this study contributes to the broader scholarship on the interplay between information and the financial markets, stemming largely from Cutler et al.’s seminal paper in 1989. [27]–[34] Recent studies have suggested trading behavior in response to news varies by investor type [35] and have examined the effects of alternate sources of news, such as Google and Yahoo! search volumes [36]–[38], Wikipedia usage [39], and Twitter mood [40], on stock returns and investor behavior. A comprehensive study of nearly 25 million news records revealed that news flows partially explain financial market volatility [41], though others have suggested a more minor role of news [42]. Intriguingly, our results showing a steeper and sustained decline in stock returns, after adjusting for normal market performance, as a result of negative announcements versus positive announcements suggest that the market may react asymmetrically to clinical R&D news.

There are several possible explanations for this asymmetry. Previous studies have found that the market reacts disproportionately unfavorably to product development failures than successes, and our abnormal returns analysis may lend credence to this behavior. One hypothesis is that negative announcements leave an imprint on investor perceptions of the company or confidence in the ability of senior management to conduct well-run clinical trials—that is, negative events may have a ‘reputational’ effect. [43] One could speculate that the market incorporates this ‘reputational’ component implicitly and explicitly into its valuation calculus, though a reputational cost *per se* stands in contrast to what may be expected by the efficient market hypothesis: market participants will and do make unemotional calculations of asset valuation. Alternately, one could argue that as the results of clinical trials are anticipated events, market participants have already factored risk-adjusted expectations about their outcomes into the stock price. Therefore, absolute differences in abnormal returns may arise due to varying probabilities for success or failure, levels of uncertainty, risk-taking, or some combination thereof.

Our study has several limitations. First, we analyzed events and market responses relating to large publicly traded U.S. pharmaceutical and biotechnology companies; as such, our results may not be generalizable to smaller biotech companies, though one could suspect that smaller companies dependent on a handful of drugs will be subject to larger fluctuations in stock price, or non-U.S. firms. Second, there are a number of issues that arise in treatment of small sample sizes. To mitigate potential bias and risk of Type I errors, we used nonparametric tests and bootstrap resampling accordingly. However, our sample was not sufficiently powered—or designed—to measure the effects of multiple predictors, and the interactions of those variables, on abnormal returns. Finally, while we sought to account for firm-level overlap in event windows, external and competitor events may have also influenced firm results. For example, there may be spillover or contagion effects from events that do not immediately relate to a firm’s R&D activities. [44], [45] Indeed, stock prices are better understood as multifactorial constructs, though the use of a short horizon is a clean method that controls some, if not most, of these potential confounding factors.

Despite these limitations, our findings are robust to a battery of sensitivity analyses, indicating that biomedical research is indeed a value driver for biopharmaceutical companies and that the results of this research are interpreted asymmetrically by the market. Although our results do not, at first glance, show detectable insider trading before positive or negative announcements, the potential for illicit transfers of information on clinical trial results remains a challenge for regulators and policymakers. Recently, a former executive of the hedge fund SAC Capital Advisors faced charges due to stock trades allegedly made on tips from clinical investigators on the results of an Alzheimer’s disease clinical trial. [46] Since 2007, nearly 100 people have been charged or sued by regulators for insider trading on internal results relating to drugs and devices, and more than one in five US insider trading cases involve healthcare stocks. [47] Further research expanding our sample and on the behavioral implications of asymmetric market valuations of R&D efforts would be warranted.

Public equity markets are often a vital source of capital for biopharmaceutical companies that invest in the costly process of research and development for novel, and potentially lifesaving, therapeutics. To the extent that access to capital and therefore market valuation of R&D efforts shape the course of scientific innovation, a better understanding of how markets react to clinical development events is needed.

## Supporting Information

Figure S1 Median CAR (–2, t) for positive and negative events using (–30, +30) reference window (robustness check). **Note**: The median CAR calculated at different times along the ± 2 trading day window are shown for positive (blue) and negative (red) events.
(PDF)

## References

[1] nnell JW, Blanckley A, Boldon H, Warrington B (2012) Diagnosing the decline in pharmaceutical R&D efficiency. Nat Rev Drug Discov 11: 191–200. 22378269 10.1038/nrd3681

[2] Getz KA, Wenger J, Campo RA, Seguine ES, Kaitin KI (2008) Assessing the impact of protocol design changes on clinical trial performance. Am J Ther 15: 450–457. 18806521 10.1097/MJT.0b013e31816b9027

[3] Munos B (2009) Lessons from 60 years of pharmaceutical innovation. Nat Rev Drug Discov 8: 959–968. 19949401 10.1038/nrd2961

[4] Pammolli F, Magazzini L, Riccaboni M (2011) The productivity crisis in pharmaceutical R&D. Nat Rev Drug Discov 10: 428–438. 21629293 10.1038/nrd3405

[5] DiMasi JA, Feldman L, Seckler A, Wilson A (2010) Trends in risks associated with new drug development: success rates for investigational drugs. Clin Pharmacol Ther 87: 272–277. 20130567 10.1038/clpt.2009.295

[6] Rang HP (2006) Drug discovery and development: technology in transition. Edinburgh: Churchill Livingstone/Elsevier.

[7] Arrowsmith J (2011) Trial watch: Phase II failures: 2008–2010. Nat Rev Drug Discov 10: 328–329. 10.1038/nrd3439 21532551

[8] Thomson Reuters (2012) CMR International Pharmaceutical R&D Factbook. Available: http://cmr.thomsonreuters.com/pdf/2012-cmr-factbook-exc_cbr-en.pdf. Accessed 2013 May 1.

[9] Senard JM, Montastruc P, Herxheimer A (1996) Early warnings about drugs-from the stock market. Lancet 347: 987–988. 8606610 10.1016/s0140-6736(96)90141-5

[10] Topol EJ, Blumenthal D (2005) Physicians and the investment industry. JAMA 293: 2654–2657. 15928288 10.1001/jama.293.21.2654

[11] Benowitz S (2002) Big business: when Wall Street and cancer research collide. J Natl Cancer Inst 94: 1352–1353. 12237278 10.1093/jnci/94.18.1352

[12] Steinbrook R (2005) Wall Street and clinical trials. N Engl J Med 353: 1091–1093. 16162879 10.1056/NEJMp058216

[13] Ferguson JR (1997) Biomedical research and insider trading. N Engl J Med 337: 631–634. 9271488 10.1056/NEJM199708283370910

[14] Chan SH, Martin JD, Kensinger JW (1990) Corporate research and development expenditures and share value. Journal of Financial Economics 26: 255–276.

[15] Sharma A, Lacey N (2004) Linking product development outcomes to market valuation of the firm: The case of the U.S. pharmaceutical industry. Journal of Product Innovation Management 21: 297–308.

[16] Shortridge RT (2004) Market valuation of successful versus non-successful R&D efforts in the pharmaceutical industry. Journal of Business Finance and Accounting 31: 1301–1325.

[17] Overgaard CB, van den Broek RA, Kim JH, Detsky AS (2000) Biotechnology stock prices before public announcements: evidence of insider trading? J Investig Med 48: 118–124. 10736971

[18] Rothenstein JM, Tomlinson G, Tannock IF, Detsky AS (2011) Company stock prices before and after public announcements related to oncology drugs. J Natl Cancer Inst 103: 1507–1512. 21949081 10.1093/jnci/djr338

[19] MacKinlay A (1997) Event studies in economics and finance. Journal of Economic Literature 35: 13–39.

[20] Campbell J, Lo A, MacKinlay A (1997) The econometrics of financial markets. Princeton: Princeton University Press.

[21] Pérez-Rodríguez JV, González López-Valcárcel B (2012) Does innovation in obesity drugs affect stock markets? An event study analysis. Gac Sanit 26: 352–359. 22244267 10.1016/j.gaceta.2011.07.028

[22] Panattoni LE (2011) The effect of Paragraph IV decisions and generic entry before patent expiration on brand pharmaceutical firms. J Health Econ 30: 126–145. 21074873 10.1016/j.jhealeco.2010.09.004

[23] Campbell C, Wasley C (1993) Measuring security price performance using daily NASDAQ returns. Journal of Financial Economics 33: 73–92.

[24] Kolari JW, Pynnonen S (2011) Nonparametric rank tests for event studies. Journal of Empirical Finance 18: 953–971.

[25] Fidrmuc JP, Goergen M, Renneboog L (2006) Insider trading, news releases, and ownership concentration. Journal of Finance 61: 2931–2973.

[26] Bailey W, Karolyi GA, Salva C (2006) The economic consequences of increased disclosure: Evidence from international cross-listings. Journal of Financial Economics 81: 175–213.

[27] Cutler DM, Poterba JM, Summers LH (1989) What moves stock prices? Journal of Portfolio Management 15: 4–12.

[28] McQueen G, Roley VV (1993) Stock prices, news, and business conditions. Review of Fin Studies 6: 683–707.

[29] Chan WS (2003) Stock price reaction to news and no-news: drift and reversal after headlines. Journal of Financial Economics 70: 223–260.

[30] Vega C (2006) Stock price reaction to public and private information. Journal of Financial Economics 82: 103–133.

[31] Tetlock P (2007) Giving content to investor sentiment: The role of media in the stock market. The Journal of Finance 62: 1139–1168.

[32] Barber BM, Odean T (2008) All that glitters: The effect of attention and news on the buying behavior of individual and institutional investors. Review of Financial Studies 21: 785–818.

[33] Dellavigna S, Pollet J (2011) Investor inattention and friday earnings announcements. Journal of Finance 64: 709–749.

[34] Engelberg J, Parsons C (2011) The causal impact of media in financial markets. Journal of Finance 66: 67–97.

[35] Lillo F, Miccichè S, Tumminello M, Piilo J, Mantegna RN (2012) How news affect the trading behavior of different categories of investors in a financial market. arXiv1207.3300.

[36] Preis T, Moat HS, Stanley HE (2013) Quantifying trading behavior in financial markets using Google Trends. Sci Rep 3: 1684. 23619126 10.1038/srep01684 PMC3635219

[37] Da Z, Engelberg J, Gao PJ (2011) In search of attention. Journal of Finance 56: 1461–1499.

[38] Bordino I, Battiston S, Caldarelli G, Cristelli M, Ukkonen A, et al (2012) Web search queries can predict stock market volumes. PLoS One 7: e40014. 22829871 10.1371/journal.pone.0040014 PMC3400625

[39] Moat HS, Curme C, Avakian A, Kenett DY, Stanley HE, et al (2013) Quantifying Wikipedia usage patterns before stock market moves. Sci Rep 3: 1801.

[40] Bollen J, Mao H, Zeng X (2011) Twitter mood predicts the stock market. Journal of Computational Science 2: 1–8.

[41] Hisano R, Sornette D, Mizuno T, Ohnishi T, Watanabe T (2013) High quality topic extraction from business news explains abnormal financial market volatility. PLoS One 8: e64846. 23762258 10.1371/journal.pone.0064846 PMC3675119

[42] Joulin A, Lefevre A, Grunberg D, Bouchaud JP (2008) Stock price jumps: news and volume play a minor role. arXiv0803.1769.

[43] Carpenter D (2010) Reputation and power: Organizational image and pharmaceutical regulation at the FDA. Princeton: Princeton University Press.

[44] Dranove D, Olsen C (1994) The economic side effects of dangerous drug announcements. Journal of Law & Economics 37: 323–348.

[45] Lang L, Stulz R (1992) Contagion and competitive intra-industry effects of bankruptcy announcements: an empirical analysis. Journal of Financial Economics 32: 45–60.

[46] Rothfeld M, Bray C, Pulliam S (2012) Trading charges reach SAC. Wall Street Journal. Available: http://online.wsj.com/article/SB10001424127887323713104578130930796204500.html. Accessed 2013 May 1.

[47] Armstrong D (2012) Drug stocks prey for insiders as industry resists change. Bloomberg. Available: http://www.bloomberg.com/news/2012-12-21/drug-stocks-prey-for-insiders-as-industry-resists-change.html. Accessed 2013 May 1.

