# Supplementary material for: Stock Market Returns and Clinical Trial Results of Investigational Compounds: An Event Study Analysis of Large Biopharmaceutical Companies
Source: PLoS One. 2013 Aug 7;8(8):e71966. doi: 10.1371/journal.pone.0071966 (PMC3737210; doi:10.1371/journal.pone.0071966)
Supplement: Figure S1 — Median CAR (–2, t) for positive and negative events using (–30, +30) reference window (robustness check). Note: The median CAR calculated at different times along the ± 2 trading day window are shown for positive (blue) and negative (red) events. (PDF) [file pone.0071966.s001.pdf]

# SUPPORTING INFORMATION

## Stock Market Returns and Clinical Trial Results of Investigational Compounds: An Event Study Analysis of Large Biopharmaceutical Companies

THOMAS J. HWANG
Harvard University, Cambridge, Massachusetts USA

**Figure S1**. Median CAR (-2, *t*) for positive and negative events using (-30, +30) reference window (robustness check)

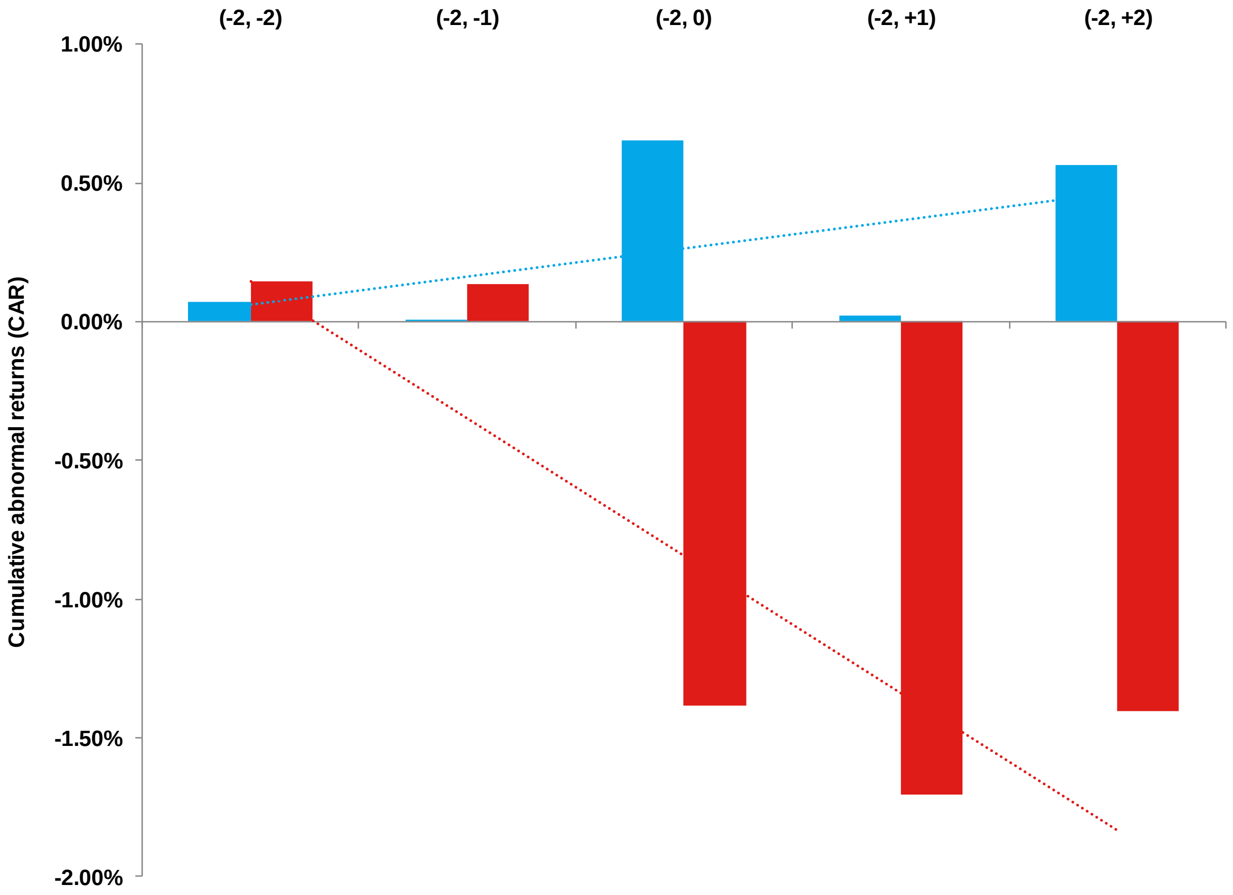


**Note**: The median CAR calculated at different times along the ± 2 trading day window are shown for positive (blue) and negative (red) events.
